# Augmented expression of superoxide dismutase 2 mitigates progression and rupture of experimental abdominal aortic aneurysm

**DOI:** 10.7150/thno.104957

**Published:** 2025-03-10

**Authors:** Huimin Yan, Ying Hu, Yang Lyu, Antonina Akk, Angela C. Hirbe, Samuel A. Wickline, Hua Pan, Elisha D.O. Roberson, Christine T.N. Pham

**Affiliations:** 1 The Department of Medicine, Division of Rheumatology, Washington University School of Medicine, Saint Louis, Missouri, USA.; 2 The John Cochran VA Medical Center, Saint Louis, Missouri USA; The Department of Medicine, Division of Rheumatology, Washington University School of Medicine, Saint Louis, Missouri, USA.; 3 The Department of Medicine, Division of Oncology, Washington University School of Medicine, Saint Louis, Missouri, USA.

**Keywords:** abdominal aortic aneurysm, oxidative stress, SOD2 mRNA, nanomedicine, proteomics

## Abstract

**Rationale:** Oxidative stress is implicated in the pathogenesis and progression of abdominal aortic aneurysm (AAA). It is suggested that an excess in reactive oxygen species (ROS) over endogenous antioxidant activities can lead to endothelial and mitochondrial dysfunction, which promotes tissue inflammation, extracellular matrix degradation, and cellular apoptosis, all pathologic features characteristic of AAA. While elevated levels of ROS in human and experimental AAA appear well established, the contribution of endogenous antioxidant systems to aneurysm formation and progression remains controversial. We demonstrate that the antioxidant enzyme superoxide dismutase 2 (SOD2), the resident mitochondrial form of SODs that protects against mitochondrial damage, is relatively deficient in established preclinical AAA. We hypothesize that augmented expression of SOD2 will protect against oxidative stress and mitigate aneurysm progression.

**Methods:** Herein, we employ a peptide-based nanoplatform to overexpress a key modulator of oxidative stress, SOD2. The efficacy of systemic delivery of murine SOD2 mRNA as an antioxidant nanotherapeutic agent was studied in two different murine AAA models. Unbiased mass spectrometry-enabled proteomics and high-dimensional bioinformatics were used to examine pathways modulated by SOD2 overexpression.

**Results:** Using two different murine models of AAA, we show that *in vivo* augmentation of SOD2 expression *via* mRNA-based nanotherapy mitigates the expansion of small aneurysms and largely prevents rupture. Mitigation of AAA is accompanied by concomitant suppression of ROS, ROS surrogate markers, and apoptotic cell death. Proteomic profiling of AAA tissue and gene set enrichment analysis show that SOD2 overexpression is associated with modulation of oxidative phosphorylation, respiratory electron transport, and fatty acid metabolism. In addition, SOD2 overexpression inhibits platelet activation, downregulates mitogen-activated protein kinase signaling, and augments levels of microRNAs miR-181a-5p and miR17-5p targets that regulate vascular inflammation and cell apoptosis, respectively.

**Conclusions:** These results confirm that SOD2 plays a pivotal role in the pathogenesis of experimental AAA and identify its potential as a therapeutic target.

## Introduction

AAA is an inflammatory disease process characterized by transmural infiltration of the aortic wall with every type of leukocytes that release matrix metalloproteinases (MMPs) and pro-inflammatory cytokines, such as tumor necrosis factor alpha (TNF-α), interleukin-1β (IL-1β), IL-6, IL-12, IL-23, interferon gamma (IFN-γ) 1, 2, leading to the degradation of extracellular matrix and acceleration of aneurysmal expansion 1, 3-6. In addition to matrix-degrading proteases and inflammatory cytokines, infiltrating inflammatory cells can produce large amounts of reactive oxygen species (ROS) that amplify oxidative stress. Studies in patients and animal models of AAA suggest that oxidative stress contributes to the production of ROS. Upregulation of nitric oxide (NO) induces the expression of MMPs in aortic endothelial cells, which exacerbates experimental AAA 7-9. Zhang *et al.* show that inducible nitric oxide synthase (iNOS) is present in human AAA and promotes tissue and cellular injury 10. Indeed, inhibition of iNOS with the selective inhibitor aminoguanidine limits experimental AAA 11. Despite promising experimental studies pointing to the pathogenic role of ROS, clinical translation has met with significant challenges due to limitations in achieving sufficient levels of antioxidant therapeutics locally at the site of AAA following systemic delivery. Indeed, no single antioxidant therapy has proven successful in clinical trials.

Endogenous antioxidant systems are enzymes that regulate oxidative stress to maintain redox homeostasis. Dysregulation of antioxidant systems resulting in an imbalance of oxidants and antioxidants and an excess of ROS production is thought to perpetuate the inflammatory cycle, activating MMPs, and inducing cellular apoptosis in AAA 12. Thus, overexpression of endogenous antioxidant enzymes as a therapeutic approach for AAA is of substantial interest. Endogenous antioxidant systems comprise a group of oxidoreductases knowns as SODs, enzymes that catalyze the conversion of superoxide anions into oxygen and hydrogen peroxide (H_2_O_2_), which is subsequently degraded to H_2_O and oxygen by catalase or other peroxidases 13. Parastatidis *et al.* show that overexpression of catalase, a peroxisomal H_2_O_2_ scavenging enzyme, *via* parenteral administration of catalase or by using a genetically engineered murine model, protects mice from CaCl_2_-induced AAA 14. On the other hand, studies on the potential involvement of SODs in AAA give contradictory results. SODs consist of three isoforms, the cytoplasmic Cu/ZnSOD (SOD1), the mitochondrial MnSOD (SOD2), and the extracellular Cu/ZnSOD (SOD3) 13. In human studies, SOD levels are significantly lower than controls in one study 15 while elevated in another 16. However, in a recent study, SOD2 is identified as a crucial or “hub” gene that may impact AAA formation through non-coding regulatory network 17. In the elastase-induced AAA model, DNA microarray confirms downregulation of antioxidant genes 18. Conversely, in another elastase-induced AAA study, aortic wall SOD2 mRNA and protein levels are found to be significantly elevated in the early days of post-elastase perfusion while SOD1 and SOD3 levels are unchanged 19. Moreover, an SOD mimetic increases MMP activity in rat aortic explants 19. Collectively, these findings identify SODs as important contributors in aneurysm development but whether their modulation represents an effective strategy in AAA management remains to be determined. Given that SOD2 plays a key role in modulating oxidative stress to protect against mitochondrial damage 20,21 and several studies suggest a relative deficiency in established AAA 15, 18, 22, we posit that SOD2 overexpression in aortic aneurysms may correct the mitochondrial dysfunction, forestalling the oxidative damage that contributes to aneurysmal expansion and rupture. To accomplish this objective, we employ a peptide-nucleic acid nanoplatform 23 to effect efficient overexpression of SOD2 in aortic tissues to explore the exact contribution of this antioxidant enzyme to AAA pathogenesis using two different experimental models.

## Results

### Relationship between oxidative stress and SOD2 expression in experimental AAA

**Figure 1:**
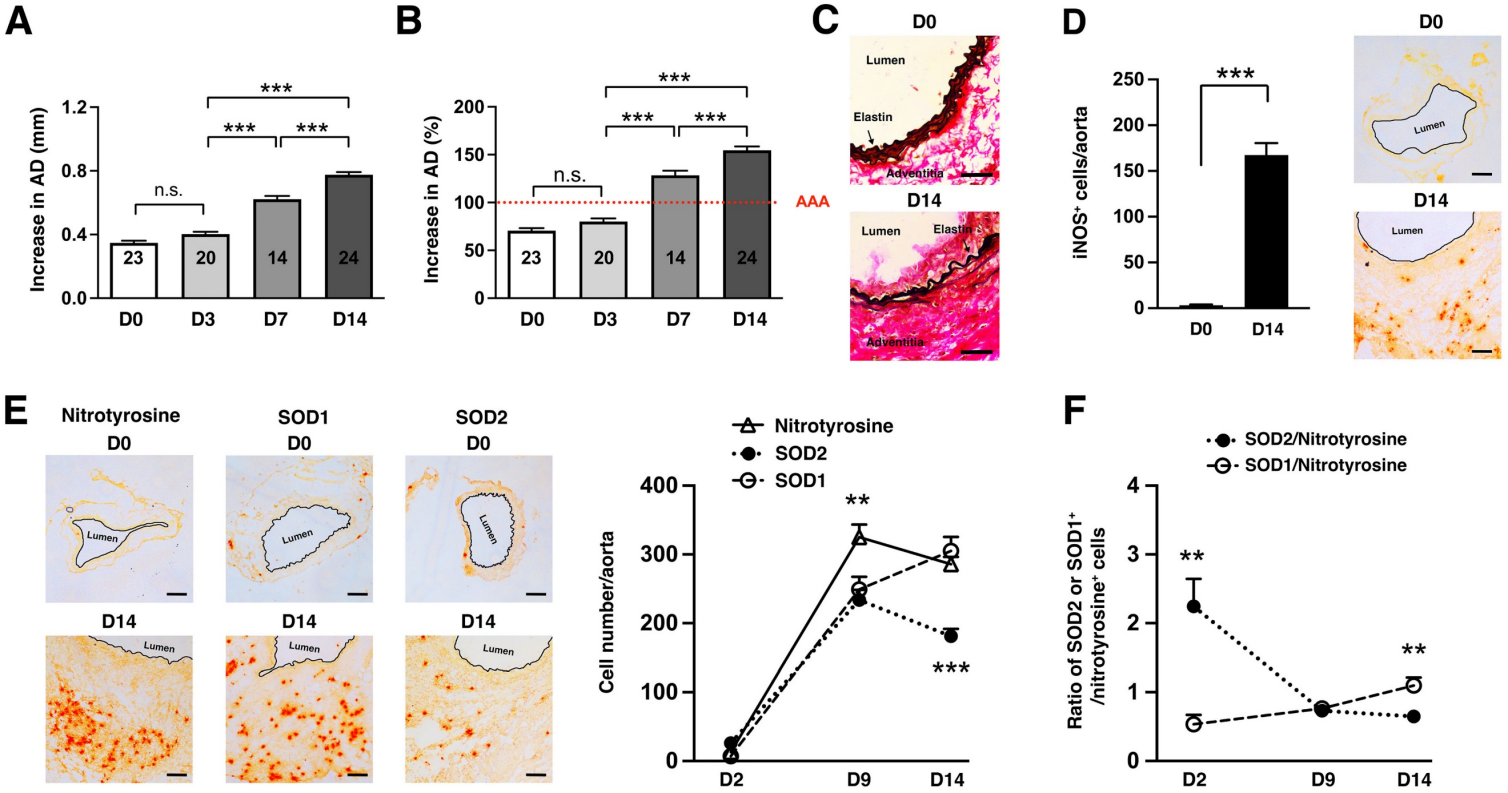
**Oxidative stress in elastase-induced AAA.** (A) Mice were transiently perfused with elastase on day 0. Aortic diameter (AD) increase is expressed in % or mm. (A) There was an immediate increase in aortic diameter (AD) of ~70% immediately post-perfusion. AAA was defined as an increase in AD of greater than 100% compared with the AD measured prior to elastase perfusion. (B) Increase in AD, measured on day 14 as mm increase. Aortic sections from day 14 were examined for elastic fiber integrity with VVG staining (C), iNOS (D) and NO/nitrotyrosine, SOD1, and SOD2 expression by immunohistochemistry (E). The number of nitrotyrosine+, SOD1^+^, and SOD2^+^ cells were enumerated over time (E) and expressed as relative ratio of SOD1/2:nitrotyrosine (F). Comparisons between multiple groups (≥3) were made by one-way ANOVA followed by Bonferroni's post-test to compare all groups of data. Comparisons between two groups were performed by two-tailed, unpaired t-test without correction. Values represent mean ± SEM derived from 4-6 non-overlapping fields per aortic section and 3-5 sections per aorta, n = 5-6 aortas per treatment. **P < 0.01, ***P < 0.001, n.s. not significant. Scale bars = 50 μm (C), 100 μm (D, E).

We first used the well-established elastase-induced AAA model in which transient porcine elastase perfusion of the infrarenal abdominal aorta on day 0 leads to aneurysmal dilatation at day 14 24. AAA is defined as an increase in the aortic diameter (AD) of more than 100% over the pre-elastase perfusion measurements 24. Elastase perfusion led to an immediate increase in AD of ~70% (Figure 1A) 25. WT C57BL/6 mice uniformly developed AAA on day 14 (mean increase in AD = 154 ± 4% or 0.78 ± 0.02 mm, n = 24) (Figure 1A-B). AAA was accompanied by characteristic fragmentation of the elastic fibers (Figure 1C). Signs of oxidative stress included marked induction of iNOS (Figure 1D) and elevated level of nitrotyrosine (a maker of ROS/NO production, Figure 1E) on day 14. We also profiled expression of nitrotyrosine, SOD1, and SOD2 over time following elastase perfusion. We observed that the number of SOD^+^ cells increased during the early phase of aneurysm development. However, the increase in SOD1^+^ cells significantly outpaced that of SOD2^+^ cells (Figure 1E). And while the number of nitrotyrosine^+^ cells continued to increase, there was progressive decrease in SOD^+^ cells after day 9 resulting in relative deficiency of SOD2 compared to SOD1 in late-stage disease (Figure 1F).

### A peptide-based nanoplatform for the overexpression of mitochondrial SOD2

**Figure 2:**
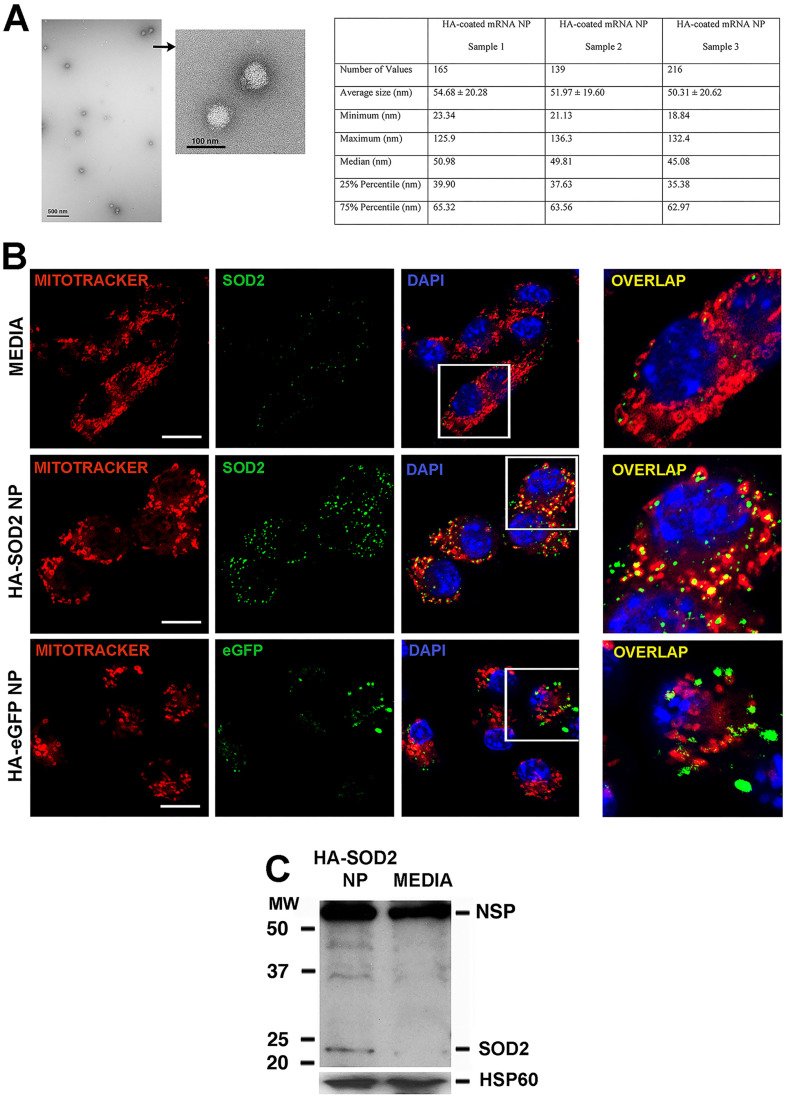
** Characterization/uptake of HA-SOD2 mRNA NP and *in vitro* expression of SOD2.** NP was prepared by mixing SOD2 mRNA (1 μg) with p5RHH (10 μmol) at 37ºC for 40 min. 5 μl of HA was added to the self-assembled NP and placed on ice for 5 min. (A) TEM of HA-SOD2 mRNA NP and sizing from 3 separate NP samples prepared simultaneously. Scale bars = 500 nm; high magnification 100 nm. (B) RAW 264.7 cells were seeded in 8-well Nunc™ Lab-Tek™ II Chamber Slide™ Glass slide System and transfected with HA-SOD2 mRNA NP or HA-eGFP mRNA NP (as irrelevant mRNA control) for 5 hours then the expression of SOD2 and eGFP was detected at 48 hours. The images were captured by a ZEISS LSM 880 confocal laser scanning microscope. Scale bars = 10 μm. (C) RAW 264.7 cells were kept in media or transfected with HA-SOD2 mRNA NP for 5 hours then harvested at 48 hours. Mitochondria were isolated according to manufacturer's directions, fractionated on SDS-PAGE, and blotted for SOD2. HSP60 served as control for protein loading; NSP = non-specific band.

Given the observed relative deficiency of SOD2 in established AAA, we hypothesized that augmentation of SOD2 expression in aortic tissue would mitigate aneurysm progression. To accomplish this objective, we turned to a peptide-based delivery platform to deliver messenger RNA (mRNA) encoding murine SOD2. We have recently shown successful *ex vivo* delivery of long mRNA sequences (> 1,000 nucleotides, nt) to cartilage explants using the p5RHH peptide-based platform 26. Building on this early success, we generated p5RHH-SOD2 mRNA NP. The SOD2 mRNA sequence was produced commercially and contained the appropriate endcaps, poly-A tail, and nucleotide base modifications (~1,000 nt) to enhance translation. The sequence also contains a mitochondrial localizing peptide component. The p5RHH-SOD2 mRNA NP was prepared by mixing 1 μg of SOD2 mRNA and 10 mmol of p5RHH peptide at 37ºC for 40 min to form a self-assembled stable and uniform NP of ~50-55 nm in diameter by TEM (Figure 2A). The NP was further functionalized with a coating of hyaluronic acid (HA), with the goal of enhancing cellular uptake *via* CD44 binding 27. The highly negative HA coating lowered the NP's zeta potential to ~ -30 mV, as previously shown 27. We showed that the HA-coated p5RHH-SOD2 mRNA NP (HA-SOD2 mRNA NP) was readily taken up by bone-marrow-derived macrophages (Figure S1 and also as previously demonstrated) 27, and the overexpression of SOD2 was readily detected in mitochondria of RAW 264.7 following *in vitro* transfection, as evidenced by colocalization with Mito tracker red (Figure 2B). The transfected RAW 264.7 cells were lysed, mitochondria isolated, mitochondrial protein lysate fractionated on SDS-PAGE and probed with a SOD2-specific antibody to further confirm overexpression (Figure 2C).

### Augmentation of SOD2 in the mitigation of elastase-induce AAA

We next administered fluorescein-labeled HA-SOD2 mRNA NP i.v. to mice on day 9 post-elastase perfusion. We observed NP localizing to the elastase-perfused abdominal aorta at 7 and 24 hours after injection (Figure S2A) with low level sequestration in the liver and minimal accumulation in other major organs (Figure S2B). Next, the fluorescein-labeled HA-SOD2 mRNA NP was administered i.v. to mice on days 5, 8, and 11 post-elastase perfusion. On day 9, 24 hours after i.v. administration NP could be observed colocalizing with MOMA-2^+^ cells in the aortic wall adventitial layer (Figure S2C). It should also be noted that it is unlikely fluorescein-labeled HA-SOD2 mRNA NP reached the aortic wall tissue mainly via cellular hitchhiking as we only detected a small percentage of monocytes (6-8%) with internalized NP in the circulation following i.v. administration (Figure S3). Moreover, repeat IV administration of NP did not affect hematologic parameters or liver/kidney function (Figure S4).

**Figure 3:**
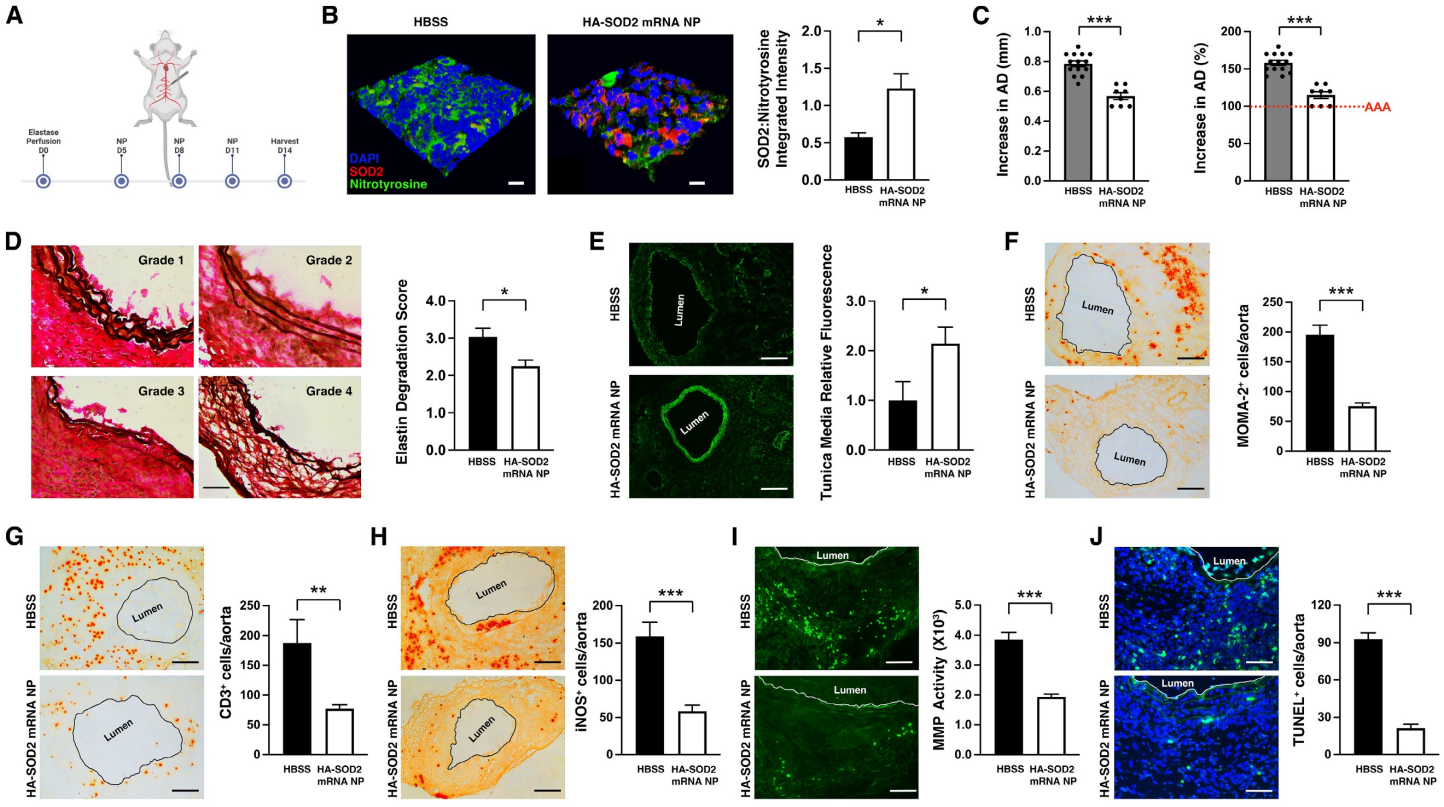
** HA-SOD2 mRNA NP in elastase-perfusion model of AAA.** (A) Mice were perfused with elastase on day 0 and administered HBSS or HA-SOD2 mRNA NP i.v. on days 5, 8 and 11 post-elastase perfusion (mRNA = 1 μg per treatment). (B) Confocal microscopy of day 14 AAA tissue in HBSS and HA-SOD2 mRNA NP treated animals. SOD2 (red), nitrotyrosine (green), DAPI (blue). Scale bars = 15 μm. Ratios of SOD2:nitrotyrosine intensity. (C) Day 14 aortic diameter (AD) increase was expressed in mm or %. (D) Histological analysis of the internal elastic laminae by VVG staining. (E) SMA content in tunica media analysis by immunofluorescence. Day 14 MOMA2^+^ (F), CD3^+^ (G), iNOS^+^ cells (H), *in situ* MMP activity (I), and TUNEL^+^ cells (J) in AAA tissue were assessed. Comparisons between two groups were performed by two-tailed, unpaired t-test without correction. Values represent mean ± SEM derived from n = 4-6 sections per aorta, n = 4-8 aortas per treatment. Scale bars = 100 μm (D), 200 μm (E, F, G and H), 100 μm (I), 50 μm (J). *P < 0.05, **P < 0.01, ***P < 0.001, n.s. not significant.

Mice were subsequently perfused with elastase on day 0 and administered HBSS or HA-SOD2 mRNA NP i.v. on days 5, 8 and 11 post-elastase perfusion (mRNA = 1 μg per treatment) (Figure 3A). NP-based mRNA delivery led to enhanced SOD2 expression while suppressing nitrotyrosine levels in aortic wall tissue (Figure 3B). HBSS had no effect on SOD2 or nitrotyrosine expression (Figure 3B). SOD2 mRNA delivery significantly mitigated AAA progression (AD = 0.57 ± 0.02 mm in HA-SOD2 mRNA NP vs AD = 0.79 ± 0.02 mm in HBSS, P < 0.001) (Figure 3C). Histological analysis showed preservation of the internal elastic laminae by semiquantitative grading (Figure 3D), consistent with higher medial smooth muscle cell (SMC) content on day 14 following SOD2 augmentation (Figure 3E). Untreated animals, on the other hand, showed higher grade of elastin degradation and reduced medial SMC content (Figure 3D, E). Mitigation of AAA was also accompanied by marked decreases in MOMA2^+^ macrophages, CD3^+^ T cells, iNOS^+^, MMP^+^ and TUNEL^+^ (apoptotic) cells (Figure 3F-J).

### Overexpression of SOD2 in the prevention of AAA rupture

**Figure 4:**
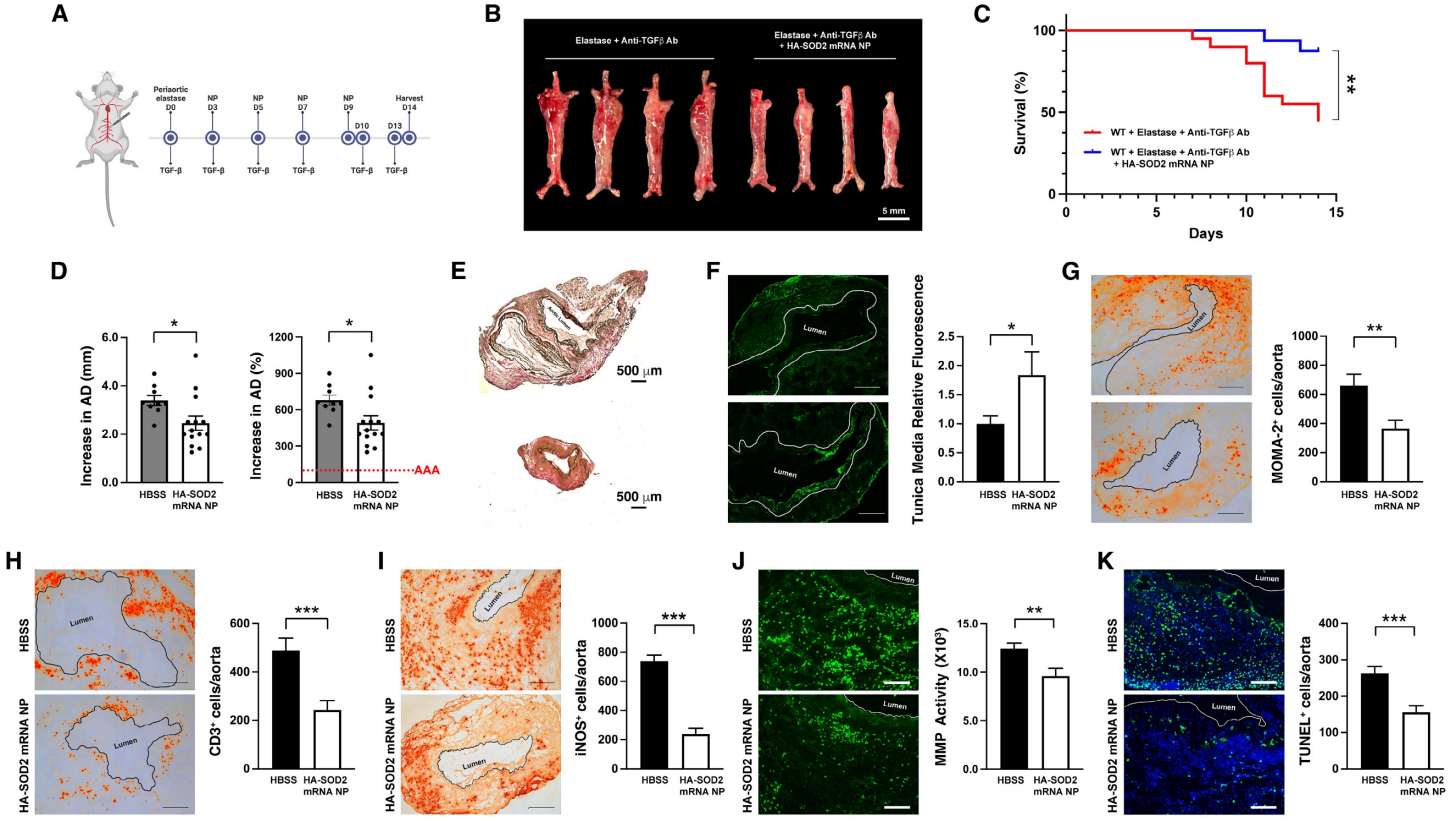
**HA-SOD2 mRNA NP in TGF-β blockade model of AAA rupture.** Periaortic elastase was applied on day 0; TGF-β antagonist and NP were administered according to the schedule shown in (A). (B) Representative macroscopic images of aortas on day 14. Scale bar = 5 mm. (C) Survival curves following the different treatment regimens. The surviving mice were sacrificed on day 14 and aortic diameter (AD) was measured. Increase in AD was expressed in mm or % (D). Representative images of the internal elastic laminae by VVG staining (E). (F) Smooth muscle actin content analysis by immunofluorescence staining; the white line outlines the area analyzed. Day 14 MOMA2^+^ (G), CD3^+^ (H) and iNOS^+^ cells (I), *in situ* MMP activity (J) and TUNEL^+^ cells (K) in AAA tissue were assessed. Comparisons between two groups were performed by two-tailed, unpaired t-test without correction. Values represent mean ± SEM derived from n = 4-6 sections per aorta, n = 4-8 aortas per treatment. Scale bars = 200 μm (F, G, H and I), 100 μm (J and K). *P < 0.05, **P < 0.01, ***P < 0.001, n.s. not significant.

To examine the role of SOD2 in AAA rupture, we turned to a TGF-β blockade model of AAA rupture, which was developed by Lareyre *et al.* 28. Peri-aortic application of elastase to the abdominal aorta combined with systemic blockade of TGF-β activity led to rapid aortic diameter expansion and aneurysmal rupture within 14 days. In this model, elastase was applied peri-adventitially to the abdominal aorta followed by systemic administration of TGF-β blocking antibody on days 0, 3, 5, 7, 10, 13 (Figure 4A), which led to marked enlargement of the abdominal aorta (Figure 4B) and ~55% rupture in our hands without intervention (Figure 4C). As described in our previous study 27, this model also exhibits signs of oxidative stress with robust level of iNOS accompanied by marked production of MMPs, aortic tissue remodeling, apoptosis and intraluminal thrombus.

**Figure 5:**
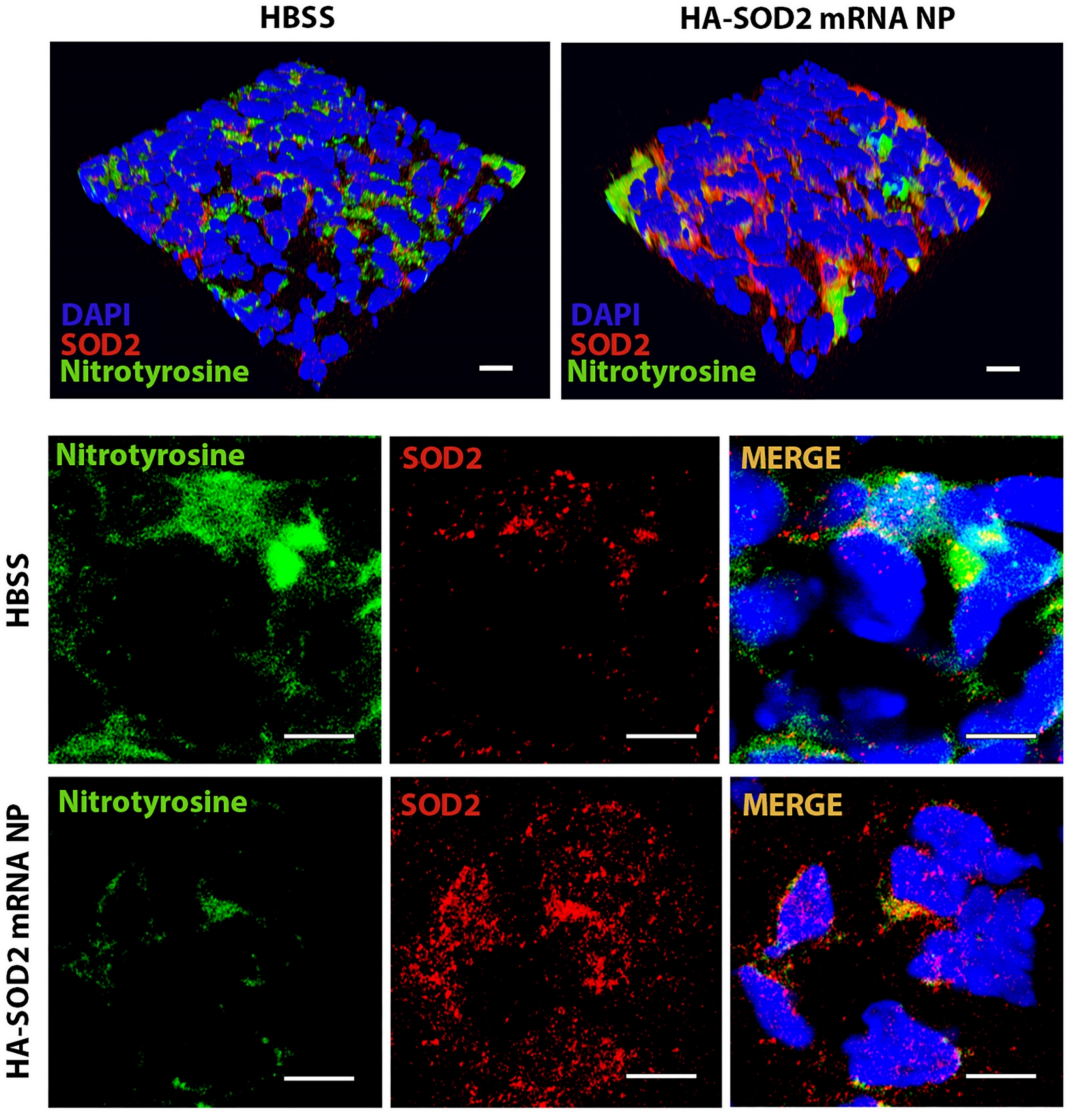
**SOD2 overexpression *in vivo* following HA-SOD2 mRNA NP administration.** Aortic sections from day 14 TGF-β blockade model of AAA rupture were examined for NO (nitrotyrosine, green) and SOD2 (red) levels. Scale bars = 100 µm (upper panel), 50 µm (lower panel).

HA-SOD2 mRNA NP administration not only delayed the onset of rupture (until day 11) but significantly protected mice from sudden death (87.5 % survival rate in treated mice, n=20 vs. 45 % survival rate in untreated mice, n=16, P < 0.01) (Figure 4C). In addition, HA-SOD2 mRNA NP treatment also mitigated the progression of AAA growth in 80% of the surviving animals (11 out of 14 surviving mice, Figure 4D). TGF-β blockade led to severe fragmentation of internal elastic laminae, formation of intraluminal thrombus, and complete loss of SMC content (Figure 4E-F). SOD2 augmentation, however, partially preserved the internal elastic laminae and SMC content (Figure 4E-F) and significantly reduced inflammatory responses in aneurysmal tissue as evidenced by decrease in MOMA2^+^ macrophages (Figure 4G) and CD3^+^ T cells (Figure 4H). Mitigation of AAA growth was also accompanied by significant reduction of iNOS^+^ cells, *in situ* MMP activity, and TUNEL^+^ cells (Figure 4I-K). Lastly, overexpression of SOD2 markedly suppressed the level of nitrotyrosine (Figure 5).

### Identification of regulatory pathways promoting AAA progression and rupture

**Figure 6:**
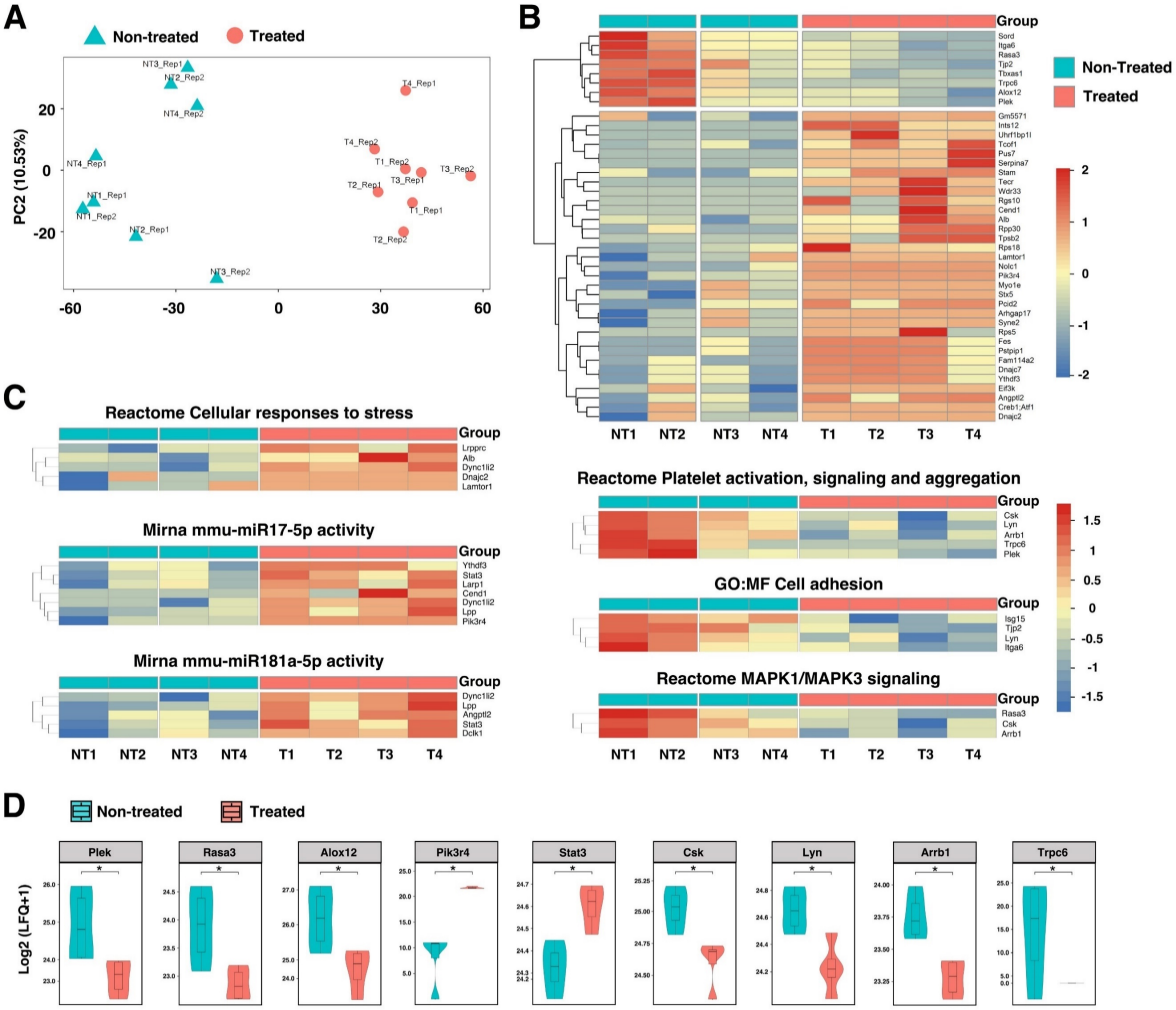
**Proteomic profiling in TGF-β blockade model of AAA following HA-coated p5RHH-SOD2 mRNA NP administration.** Aortic tissue from non-treated (NT=4) and HA-SOD2 mRNA NP-treated (T=4) were subjected to unbiased mass spectrometry-enabled proteomics and high-dimensional bioinformatics. (A) PCA plot distinguished the non-treated and treated samples based on gene expression profiles. (B) Hierarchical cluster analysis showing relative abundances of proteins in non-treated (NT) and HA-SOD2 mRNA NP-treated (T) aortas. (C) Heatmaps of significantly enriched pathways. (D) Enhancement of key protein components following HA-SOD2 mRNA NP treatment. *P < 0.001.

To further identify proteins and molecular pathways that contribute to AAA progression and rupture, we turned to unbiased mass spectrometry-enabled proteomics and high-dimensional bioinformatics to provide the complete proteome profiling of HA-SOD2 mRNA NP-treated mice compared to non-treated animals. We analyzed non-imputed data to arrive at the complete proteome profiles of 4 HA-SOD2 mRNA NP-treated (T1-4) and 4 non-treated abdominal aortas (NT1-4) of day 14 surviving animals from the TGF-β blockade model. Principal Component Analysis (PCA) was conducted to assess sample distribution and distinguish the non-treated vs. treated samples based on their protein abundance/expression profiles (Figure 6A). A heatmap of differentially expressed proteins is shown in Figure 6B. The upper split revealed that NT1 & NT2 have high detection while NT3 and NT4 have medium detection (Figure 6B); treated T1-4 have low detection, (Figure 6B). Compared with treated aortas, which have an average AD of 3.25-3.30 mm, NT1 and NT2 have an average AD of 4.00-4.50 mm and exhibited localized hemorrhage, suggesting impending rupture. Thus, the high detection of Plex (pleckstrin), Trpc6 (transient receptor potential channel 6), Rasa3 (Ras p21 protein activator 3), and Alox12 (arachidonate 12-lipoxygenase) may portend risk of arterial aneurysm rupture while overexpression of SOD2 consistently downregulated these pathways, potentially protecting the animals from aneurysm rupture and sudden death. Subsequent analysis of significantly enriched pathways derived from AAA proteomes revealed that HA-SOD2 mRNA NP treatment modulated cellular response to stress, potentially through the regulation of microRNA (miR)17-5p, which inhibits apoptosis 29, 30, and miR 181a-5p, which regulates vascular inflammation/endothelial cell senescence via STAT3 signaling pathway (Figure 6C-D) 31, 32. Furthermore, HA-SOD2 mRNA NP treatment inhibited pathways involved in platelet activation/aggregation (Csk, Lyn, Arrb1, Plek), cell adhesion (Isg15, Lyn), and MAPK1/3 signaling (Rasa3, Csk, Arrb1) (Figure 6C-D). An expanded list of the pathways modulated by SOD2 overexpression can be found in Figure S5.

**Figure 7:**
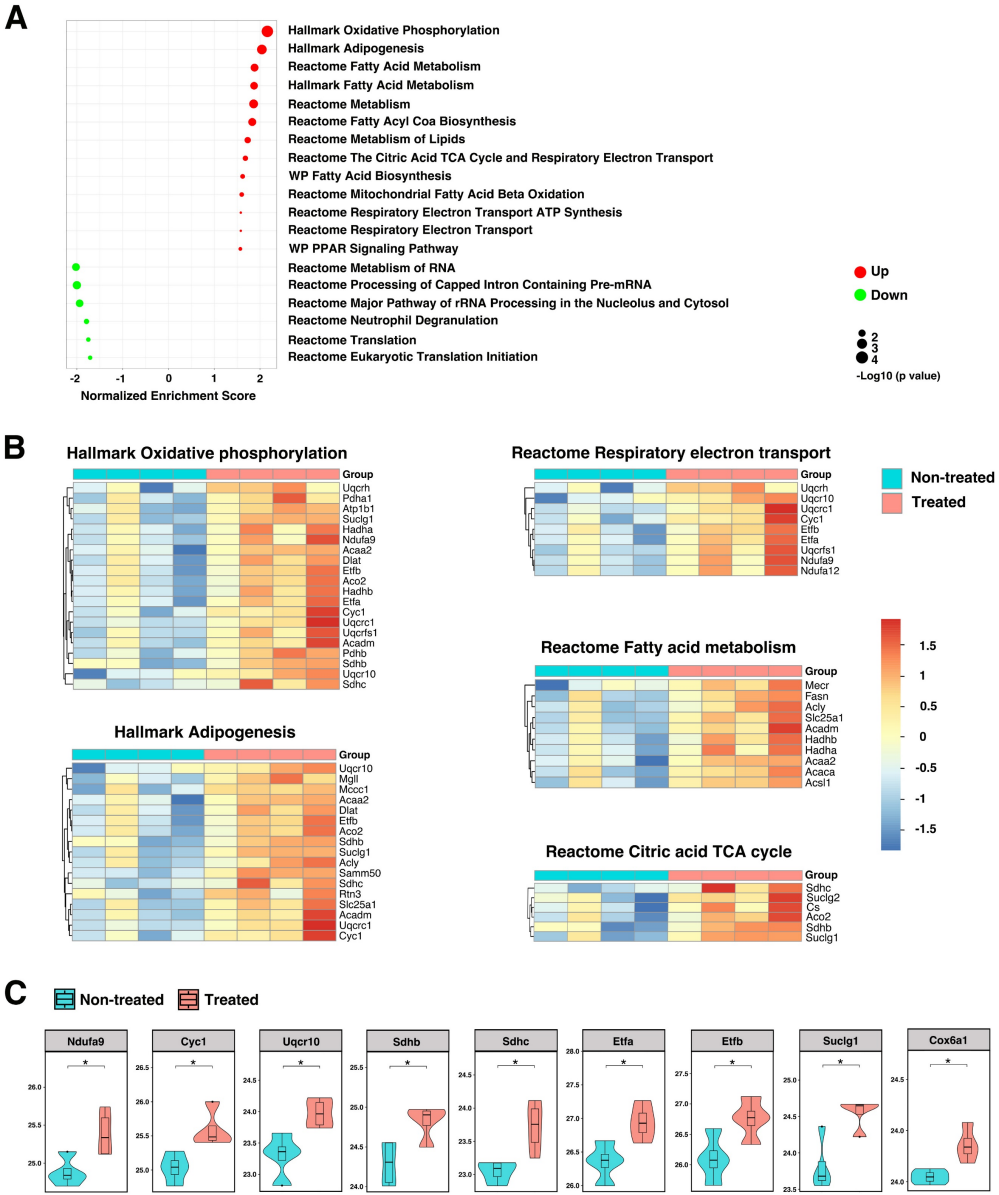
**Contribution of SOD2 in the maintenance of mitochondrial redox balance**. (A) GSEA revealed significantly enriched pathways in mitochondria following SOD2 augmentation in TGF-β blockade model of AAA. Heatmaps (B) and enhancement of key protein components (C) of pathways that control oxidative-phosphorylation, respiratory electron transport, fatty acid metabolism, and TCA cycle. *P < 0.05.

Because the formation and progression of AAA is usually accompanied by mitochondrial dysfunction and oxidative stress, we sought to further explore molecular pathways and identify effectors participating in mitochondrial biogenesis and the generation of mitochondrial ROS. The majority of ROS (about 90%) is generated during the mitochondrial oxidative phosphorylation process as byproduct. Excessive production of ROS can lead to mitochondrial dysfunction and cell death. Analysis of imputed data revealed significant enrichment in pathways associated with oxidative phosphorylation, lipid metabolism, citric acid tricarboxylic acid (TCA) cycle, and respiratory electron transport in TGF-β blockade, AAA rupture model (Figure 7A-B and Figure S6). The oxidative phosphorylation system consists of five protein complexes, of which protein complexes I - IV are respiratory chain complexes, including NADH dehydrogenase (NADH-ubiquinone oxidoreductase; complex I), succinate dehydrogenase (complex II), cytochrome c oxidoreductase (cytochrome b and c1; complex III), and cytochrome c oxidase (complex IV) 33. Enhancement of key protein components of the oxidative phosphorylation system (Ndufa9, Cyc1, Uqcr10, Sdhb/c) were observed after HA-SOD2 mRNA NP treatment (Figure 7C). Proteins in the citric acid TCA/respiratory electron transport systems that were augmented with HA-SOD2 mRNA NP treatment include Etfa/b, Suclg1, Cox6a1 (Figure 7C). Additionally, we observed significant enhancement of Slc25a1, Acadm, Hadha/b, Acaa2 in the fatty acid/lipid metabolism pathways with SOD2 augmentation (Figure S6). Conversely, we observed decrease in proteins associated with transcription/translation and neutrophil degranulation in animals treated with HA-SOD2 mRNA NP (Figure 7A).

## Discussion

The exact contribution of SOD2, the most abundant isoform of SOD found in mitochondria, to AAA pathogenesis has so far remained undefined although there are contradictory published data in human and experimental models 15, 18, 19. In normal cellular function, the redox status reflects a homeostatic balance between oxidants and antioxidants, preventing excessive generation of ROS that can contribute to cellular damage and the pathophysiology of several diseases. Oxidative stress is also believed to play an important role in AAA pathogenesis by destabilizing mitochondrial membrane potential and redox transition, which have deleterious effects on mitochondrial integrity and cellular viability. Our preclinical data show that SOD2 rapidly rises in the early phase of AAA development, which likely represents the host's defense mechanism in response to ROS generated by oxidative stress. However, this early increase is insufficient to oppose the inflammatory responses in AAA and relative deficiency of SOD2 in the late-stage disease further promotes aneurysmal expansion. Excessive ROS, particularly within mitochondria, can lead to tissue destruction through various pathways including NF-κB, which functions as a molecular switch linking ROS to inflammation in many chronic disease states 34. Persistent oxidative stress also leads to the production of inflammatory mediators (i.e. cytokines, chemokines, MMPs) that recruit inflammatory cells including macrophages and T cells. Thus, the decrease in immune cells following SOD2 augmentation likely reflects a decrease in inflammatory stimuli. Our findings that augmented expression of SOD2 mitigated aneurysm progression and rupture in experimental AAA elucidated the pivotal role of this antioxidant enzyme and revealed additional regulatory pathways downstream of its activities.

SOD2 efficiently converts superoxide to the less reactive hydrogen peroxide (H_2_O_2_), which can then freely diffuse across the mitochondrial membrane to undergo breakdown by cytoplasmic peroxidases such as catalase to combat the damaging effects of ROS 35. Increasing evidence suggests a key role for mitochondrial damage due to oxidative stress driving AAA pathophysiology 36. Moreover, genetic variations in the ROS-generating NADPH oxidase genes have been linked to higher risk of AAA rupture 37. Mitochondrial proteins are primary targets for oxidation, causing proteome imbalance that further exacerbates oxidative stress 38. Dysfunctional mitochondria due to excessive ROS has been associated with many disorders 39, including cardiac diseases 40, neurodegenerative diseases 41, and aging 38. However, the exact role of mitochondrial antioxidant SOD2 in the progression and rupture of AAA remains untested until now. Herein we showed that SOD2 overexpression in mitochondria mitigated the progression of AAA likely by modulating pathways that control oxidative phosphorylation, respiratory electron transport, fatty acid metabolism, and TCA cycle kinetics. Mitigation of AAA progression and redox modulation was also reflected in the downregulation of platelet activation, MAPK signaling, restriction of vascular inflammation via miR-181a-5p activity, and inhibition of cellular apoptosis *via* miR17-5p activity and STAT3 signaling. Of note, these miRs were previously identified as being differentially expressed in AAAs 17.

AAA rupture represents a true medical emergency as it is associated with a mortality rate of ~81% 42. The ability to predict AAA rupture, however, remains an unmet medical need. Currently, the most widely used criterion for prediction of rupture remains the maximum diameter of AAA 43. Although other factors such as aortic stiffness have been evaluated 44, further validation in larger cohorts is still needed. The rupture of an aneurysm is not a sudden event but rather the culmination of many small occurrences (i.e. microleaks) that lead to serious and potentially fatal consequences. Our proteome profiles revealed enhanced concentrations of Plex, Trpc6, Rasa3, Alox12 in non-treated aortas that exhibited localized hemorrhage. Pleckstrin (Plex) is a platelet protein that, upon platelet activation, translocates to the plasma membrane where it is phosphorylated by the protein kinase C (PKC), exerting downstream effects on platelet activation 45, and secretion of platelet factors that contribute to thrombus formation 46 and the risk of rupture 47. Trpc6 regulates a multitude of physiologic processes including vascular structural integrity and permeability 48. Trpc6 also has a direct role in leukocyte extravasation and endothelial inflammatory response and knockdown of Trpc6 blocks leukocyte transmigration 49. Recent data revealed that RASA3, which belongs to the GAP1-family GTPase-activating proteins (GAPs), can act as gatekeeper of T cell activation and adhesion 50. Although current evidence supports a pro-inflammatory role of T cells in the progression of AAA 51, targeted therapy against T cells in AAA presents significant challenges. Thus, GAPs may offer an alternative T cell targeting strategy. Lipid peroxidation induced by ROS plays a crucial role in various cell death processes, including apoptosis, autophagy, and ferroptosis 52. ALOX12 catalyzes the addition of molecular oxygen to arachidonic acid and its activation can enhance lipid ROS accumulation, promoting ferroptosis, which has been implicated in many conditions, including AAA 53-56. Taken together, our proteomic analysis identifies a set of proteins that may be explored as markers of impending AAA rupture.

Currently there is no selective or molecularly targeted therapy for AAA. Patients may be receiving medications for blood pressure control or lipid lowering that may exert supportive benefit. Open surgical repair or minimally invasive endovascular repair with catheter delivered grafts can be utilized at later stages of the disease to prevent catastrophic rupture and maintain aortic patency. Molecular targeted NP-based nanomedicine offers a promising translational approach to mitigate AAA progression and rupture prevention. Nanotechnology-based therapy for AAA treatment is beginning to emerge and promises to overcome challenges presented by systemic delivery of antioxidants 57. In this study, we employed a peptide-based NP structure that enables rapid nucleotide polyplex self-assembly, cellular uptake, prominent endosomal permeabilization and coordinated release of siRNA or mRNA into cytoplasm 58, 59. We also functionalized the peptide-siRNA/mRNA NP with a HA coating to enhance macrophage uptake 26, 27.

The delivery of mRNA into cells has been attempted using a variety of platforms, including lipid-based nanocarriers. The recent success of COVID-19 vaccines illustrates the potential of mRNA technologies to revolutionize medical care. We have shown previously that delivery of p5RHH-WNT16 mRNA NP to human cartilage explants led to the expression of its downstream effector molecule lubricin, which is important for cartilage protection 26. Herein, we demonstrated that the HA-SOD2 mRNA NP effectively suppressed elastase-induced AAA, confirming that the delivery of mRNA using our p5RHH platform also works effectively *in vivo*. We have also previously shown that the p5RHH-siRNA NP has a favorable safety profile *in vivo*, even after repeated injections 60, 61. Likewise, we showed in this study that the delivery of HA-SOD2 mRNA NP did not lead to sustained accumulation of the NP in major organs or affect hematologic parameters or liver/kidney function. It should be noted that, unlike previous interventions in which antioxidant therapy was initiated prior to or at time of AAA induction, SOD2 mRNA was delivered after small AAA was established in our studies. Moreover, early intervention largely prevented rupture, suggesting sustained reduction in aortic oxidative stress with this approach.

In summary, proteomic profiling and analysis of aortic tissue following augmentation of SOD2 expression revealed enrichment in pathways related to inflammation/inflammatory responses (i.e., STAT3/MAPK signaling), platelet activity, and translation/transcription in addition to oxidative phosphorylation and neutrophil degranulation, pathways that are predominant in human aneurysms 62. Moreover, we found that miR181 and miR17 activities are modulated by SOD2 and these miRs may be tracked as biomarkers of disease progression in the circulation. Thus, these findings shed further insights to the contribution of SOD2 in the maintenance of vascular redox balance and revealed several potential therapeutic targets downstream of SOD2 that may be explored as therapeutic targets in the mitigation of AAA progression and rupture. Furthermore, we have established the efficiency of peptide-based delivery of mRNA for AAA treatment that can be extended to the modulation of a multitude of pathways in myriad chronic disease states. Finally, the promising findings suggest that molecularly targeted nanotherapeutic approach for the management of small AAA warrants further exploration and validation.

### Limitations

We have examined the contribution of SOD2 in two different models of AAA, a non-rupture and rupture model. Whether the role of SOD2 can be generalized to all AAA preclinical models was not explored. Moreover, whether *in vivo* SOD2 augmentation could be successfully translated into clinical application still awaits further study.

## Materials and Methods

### Study design

This study aimed to investigate the potential of enhanced expression of SOD2 as antioxidant therapy through peptide-based delivery in the development and progression of AAA. For *in vivo* study, two different murine models of AAA were used, and the number of mice was determined based on previous experience with these rodent models of AAA to detect a statistically significant differences at 80% power and 5% significance. Mice were randomly assigned to experimental groups, and the surgeon was blinded to group assignment. The mice were excluded with obvious technical problems during procedure. The *in vitro* experiments were blinded when possible and sample sizes were determined to be appropriate for determining the effect of each procedure. Techniques and assays included flow cytometry, immunostaining, Western blot, mass spectrometry and various other molecular and cellular assays. Further method details are available as follows. For all experiments, the number of replicates, statistical tests used, and P values are described in the figures and legends.

### Animals

WT C57BL/6J male mice (Cat# 000664) were obtained from the Jackson Laboratory (Bar Harbor, ME). All animal experiments were performed in compliance with guidelines and protocols approved by the Division of Comparative Medicine at Washington University in St. Louis. The animal protocol is subjected to annual review and approval by The Animal Studies Committee of Washington University.

### p5RHH-mRNA nanoparticles (NP) preparation and characterization

p5RHH peptide (provided by Genscript) was dissolved at 20 mM in DNAse-, RNAse-, and protease-free sterile purified water (Cellgro) and stored in 5 µL aliquots at -80°C before use. 10 mg Sodium Hyaluronate (HA, Cat# HA1M-1, Lifecore Biomedical) was dissolved in 1 mL HBSS with Ca^2+^ and Mg^2+^ (Gibco, Life Technologies) by sonification for 1 hour and then ultracentrifuge at 90,000g for 40 min. The supernatant was aliquoted at 50 µL and stored at -80°C until use.

The p5RHH-mRNA NP was prepared as follows: 1 µg of eGFP mRNA (TriLink Biotechnologies, San Diego, CA, USA), or 1 µg of mouse SOD2 mRNA (TriLink Biotechnologies, San Diego, CA, USA) were added to 98 mL HBSS with Ca^2+^/Mg^2+^ then mixed well. 0.5 µL of p5RHH peptide (10 mmol) was added, mixed well and the mixture was incubated at 37°C for 40 min. After incubation, 5 µL of HA was added to the mixture and placed on ice for 5 min prior to use.

Nanoparticle size was measured by transmission electron microscope (TEM, performed by the Washington University Center for Cellular Imaging, https://wucci.wustl.edu/) and by dynamic light scattering (DLS) using NanoBrook Omni (Brookhaven) and reported on a number basis for comparison with TEM imaging. Zeta potential was measured by Phase Analysis Light Scattering (PALS) by using NanoBrook Omni (Brookhaven). The TEM images were loaded onto ImageJ and the average size was acquired based on 3 independent NP samples and 139-216 particles were measured per sample.

### Expression of SOD2 in mitochondria *in vitro*

HA-coated p5RHH-SOD2 mRNA NP was prepared as described above. RAW 264.7 cells were cultured in DMEM with 10% fetal bovine serum (FBS) at 37°C and seeded at 8.0×10^5^/well in 6-well-plate, 5 plates for each treatment. The cells were starved with DMEM without FBS for 30 min, then transfected with HA-SOD2 mRNA NP or HA-eGFP mRNA NP (as irrelevant mRNA control) in Opti-Minimum Essential Medium (MEM) (100 µL NPs + 900 µL Opti-MEM) for 5 hours. Untransfected control cells were kept in Opti-MEM for 5 hours. The cells were then rinsed with PBS and cultured in DMEM with 10% FBS for 48 hours. The cells were harvested and 2.0×10^7^ cells per treatment were used for mitochondria isolation by non-mechanical, reagent-based method according to the manufacturers (Mitochondria Isolation Kit, Cat# 89874 Thermo Scientific). The mitochondrial samples were fractionated by SDS-PAGE, transferred to PVDF, and the membrane was blotted with anti-SOD2 Ab (Cat# bs-1080R, Bioss Antibodies) and anti-Hsp60 Ab (Cat# 4870S, Cell Signaling Technology). Bands were visualized using a Super Signal Chemiluminescent substrate Kit (Cat# 34080, Thermo Scientific).

### Mitochondrial staining

RAW 264.7 cells were seeded at 1.0×10^5^/well in 8-well Nunc™ Lab-Tek™ II Chamber Slide™ Glass slide System (Cat#:177402 Thermo Fisher) in DMEM with 10% FBS at 37°C. The cells were starved for 30 min prior to culture in Opti-MEM (Thermo Fischer Scientific) containing HA-SOD2 mRNA NP at 37°C for 5 hours. Then the cells were rinsed and cultured in DMEM with 10% FBS. After 48 hours, the cells were incubated with Mito Tracker™ Dyes for mitochondria labeling (Cat#: M7512, Thermo Fischer Scientific) at 37°C for 15 min, then fixed with 4% paraformaldehyde in PBS, permeabilized with 0.1% Triton X-100/PBS, and blocked with 8% BSA. Anti-mouse SOD2 antibody (1:100 dilution, Cat#: bs-1080R, Bioss Antibodies) was applied overnight. The mitochondria were visualized using AiryScan Super Resolution Imaging with ZEISS LSM 880 Confocal Laser Scanning Microscope.

### Murine elastase-induced AAA

AAA was induced as previously described 25. Only male mice were used in these studies, as AAA is a disease with strong male predominance. Briefly, 8- to 10-week-old male wildtype (WT) C57BL/6J mice were anesthetized with an i.p. injection of ketamine (87 mg kg^-1^), xylazine (13 mg kg^-1^), and acepromazine (2 mg kg^-^1) KXA cocktail. For post-operative pain control mice were administered Buprenorphine-SR (0.5 mg kg^-^1) one hour prior to surgery and the effect was expected to last 72 h. Immediately prior to surgery, lidocaine (0.5%) was injected into the subcutaneous space below the planned incision line. A laparotomy was then performed under sterile conditions. The infrarenal aorta, from the left renal vein to the aortic bifurcation was isolated, ligated and perfused for 5 min and 30 secs with a solution containing 0.145 U/mL type 1 porcine pancreatic elastase (Cat# E-1250, Sigma-Aldrich, St Louis, MO) via infusion pump. The tie was released, and the maximal post-perfusion aortic diameter was measured with a calibrated ocular grid and the aortotomy closed. The animals were randomly assigned to receive NP treatment or HBSS. WT mice were injected i.v. with HA-SOD2 mRNA NP on day 5, 8 and 11 and control mice received HBSS. The number of mice per genotype per treatment is indicated in the figure legends. On day 14, unless otherwise indicated, a second laparotomy was performed following anesthesia with KXA cocktail, and the aortic diameter measured prior to euthanasia and tissue procurement.

### TGF-β (Transforming Growth Factor-β) blockade model of AAA rupture

As previously described 28, the mouse AAA rupture model was induced by combined topical application of elastase on the abdominal aorta with systemic blockade of TGF-β activity using a neutralizing mouse monoclonal anti-TGF-β. The solution of pancreatic porcine elastase (Cat# E-1250, Type I, ≥4.0 units/mg protein, Sigma-Aldrich, St Louis, MO) was filtered through a 0.22 µm filter. Briefly, a laparotomy on C57BL/6J male WT mice (8- to 10-week-old) was performed under sterile conditions. After anesthesia and analgesia as described above, the abdominal aorta from below the left renal vein to the iliac bifurcation was isolated *in situ* circumferentially after careful dissection. 10 µL of active pancreatic porcine elastase was topically applied onto the exposed segment of abdominal aorta for 10 min. The diameter was measured *in situ* after a 10 min-application of elastase. The mice were injected i.p. 3 times/week with 250 mg of the anti-mouse TGF-β blocking antibody (BioXcell, Cat#:BE0057, clone 1D11.16.8) starting on the day of the surgery and until day 13 after surgery. The animals were randomly assigned to receive NP treatment or HBSS. On day 3, 5, 7 and 9 after surgery, HA SOD2 mRNA NP was administered i.v. to treatment group and HBSS to control group. The entire infrarenal aorta was harvested on day 14 and analyzed by histology.

### Verhoeff-van Gieson (VVG) staining

VVG staining was performed on OCT-embedded 9 µm frozen cross-sections of mouse AAA tissue using the Elastic Stain Kit (Cat#: HT25A, Sigma-Aldrich). Briefly, the cross-sections were fixed with 4% PFA and stained in Working Elastic Stain Solution for 10 min and differentiated in Working Ferric Chloride Solution. The sections were dehydrated with Xylene and mounted. The images were acquired with a Leica Digital Microscope 2000 with a Leica DMC 4500 camera using Leica Application Suite (LAS) X software.

### Immunohistochemistry and immunofluorescence

OCT-embedded 9 µm aortic frozen cross-sections of AAA tissue were used for

Immunohistochemical staining. Biotin anti-mouse CD3 (1:100 dilution; Cat# 553060, BD Biosciences), rabbit anti-mouse iNOS (1:100 dilution; Cat# ab3523, Abcam, Cambridge, MA), rat anti-mouse MOMA-2 (1:200 dilution; Cat# ab33451, Abcam, Cambridge, MA), mouse anti-mouse nitrotyrosine antibodies (1:100 dilution, Cat#: 189542, Cayman Chemical), rabbit anti-mouse SOD1(1:100 dilution, Cat#: orb186008, Biorbyt) or SOD2 (1:100 dilution, Cat#: bs-1080R, Bioss Antibodies) primary antibodies were applied to the sections for 1 hour at RT followed by the appropriate HRP-conjugated secondary antibodies. Enumeration of positive cells were performed by an experimenter blinded to the treatment. Mouse anti-mouse α-smooth muscle actin (1:200 dilution; Cat# A5228, Sigma-Aldrich) were visualized with a donkey-anti mouse FITC-secondary antibody. Data presented was derived from 6-9 serial cross-sections that spanned the entire abdominal aorta, with 5-8 aortas per treatment.

### Confocal microscopy of AAA tissue

The frozen cross-sections were fixed and permeabilized with 0.05% Triton X-100/PBS, then blocked in 8% BSA and incubated with rabbit anti-mouse SOD2 antibody (1:100 dilution, Cat#: bs-1080R, Bioss Antibodies) and mouse anti-mouse nitrotyrosine antibodies (1:100 dilution, Cat#: 189542, Cayman Chemical) at RT for 1 hour. Rhodamine (TRITC) conjugated anti-rabbit (Cat#: 711-025-152, Jackson ImmunoResearch) or FITC conjugated anti-mouse secondary antibodies (Cat#: 715-096-150) were applied. Nuclei were counterstained with DAPI. All images were visualized and acquired with ZEISS LSM 880 Confocal Laser Scanning Microscope. Images were loaded into Image J software (http://rsb.info.nih.gov/ij) for analysis. The quantification of the expression of SOD2 and nitrotyrosine was presented as mean integrated density (IntDen). The mouse data was obtained from 4-6 non-overlapping fields per aortic section and 3-5 sections per aorta, 5-6 aortas per treatment.

### *In situ* zymography

Gelatinase activity was measured on 9 µm unfixed frozen sections by incubation in DQ Gelatin (25 mg/mL; Cat # 12054; Invitrogen Molecular Probes) for 1 h at room temperature. 25 µmol/L EDTA was set for negative control. All zymographic images were acquired on a LEICO DM 2000 microscope attached with Digital Microscope Camera Leica DMC4500 and acquired with Leica Application Suite (LAS) X software. The quantification of the gelatinase activity was performed with software Image J and presented as mean IntDen. Quantification was performed by an experimenter blinded to the treatment. Data were analyzed from 6-9 cross sections per aorta, with 5-8 aortas per treatment.

### TUNEL assay

An apoptosis assay was performed to identify DNA fragmentation associated with terminal deoxynucleotidyl transferase-mediated dUTP nick-end labeling (TUNEL). Detection of apoptotic cells was performed on day 14 non-fixed frozen aortic sections using an *In situ* Cell Death Detection Kit (Cat#: 11-684-795-910, Roche). Briefly, the aortic sections were rinsed with PBS, then permeabilized with 0.5% TWEEN-20/PBS for 15 min and blocked with 8% BSA solution. TUNEL reaction mixture, freshly prepared according to the manufacturer's protocol, was applied to aortic sections for 1 hour at 37 °C, rinsed 3 to 5 times with PBS, and mounted with VECTASHIELD mounting medium containing DAPI (Cat#: H-1200, Vector Laboratories). The TUNEL^+^ cell number cells were enumerated across the entire aortic section. Enumeration of TUNEL^+^ cells was performed by an experimenter blinded to the treatment. Data represent 5-8 sections per aorta, 6-8 aortas per treatment.

### Hematologic parameters and serum chemistries

HA-SOD2 mRNA NP was administrated to mice on day 5, 8 and 11 post-elastase perfusion. On day 14, the mice were euthanized, and blood was collected from the inferior vena cava. CBC, differentials and serum chemistries (hepatic and renal functions, including AST, ALT, BUN, Creatinine and ALKP) were assessed. Analysis was performed by the Washington University Department of Comparative Medicine.

### IVIS imaging

HA-SOD2 mRNA NP was prepared according to the methods above. 5 µL of fluorescein labeled-HA (10 mg/mL, Cat# F1177, Sigma-Aldrich) was added to the mixture and incubated on ice for 5 min. WT mice on day 9 of surgery were injected i.v. with fluorescein-labeled HA-SOD2 mRNA NP. The mice were sacrificed and aorta, heart, liver, spleen, lung, kidney, bladder was harvested 7 h or 24 h after injections*. In vivo* fluorescent images were acquired with In-Vivo Multispectral FX Pro imaging system and analyzed with Bruker MI SE 7.1 software (Bruker BioSpin Corporation). The settings (excitation, 650 nm; emission, 700 nm; exposure time, 30 s) were used for image acquisitions at various time points after i.v. injection of NP. After scanning, the aortas were frozen and cross-sectioned at 9 µm. Rat anti-mouse MOMA-2 (1:200 dilution; Cat# ab33451, Abcam, Cambridge, MA) primary antibodies were applied to the sections followed by the Rhodamine (TRITC) conjugated secondary antibodies. Nuclei were counterstained with DAPI. All images were visualized and acquired with ZEISS LSM 880 Confocal Laser Scanning Microscope.

### *In vitro* NP uptake by macrophages

RAW 264.7 cells were cultured in DMEM with 10% FBS at 37°C. Cells were seeded at 1.0×10^5^/well in 8-well Nunc™ Lab-Tek™ II Chamber Slide™ Glass slide System (Cat#:177402 Thermo Fisher). The cells were starved for 30 min prior to culture in Opti-MEM (Thermo Fischer Scientific) containing fluorescein-labeled HA-SOD2 mRNA NP at 37°C for 4 hours after which the cells were rinsed with PBS, fixed with 4% paraformaldehyde in PBS and examined for NP uptake. F-actin (Cat# T7471, 1:200 dilution, Invitrogen at Thermo Fischer Scientific) was added to the cells prior to mounting with Vectashield containing DAPI. The images were captured by a ZEISS LSM 880 confocal laser scanning microscope.

### *In vivo* NP uptake by peripheral blood cells

Fluorescein-labeled HA-SOD2 mRNA NP or HBSS were administrated i.v. to WT mice on day 9 post-elastase perfusion. After 30 min or 1 hour, the mice were euthanized and whole blood was collected from the inferior vena cava. The peripheral white blood cells were isolated and analyzed by flow cytometry. The percentage of monocytes, lymphocytes and neutrophils that had taken up NP intracellularly was assessed using *BD CellQuest Pro* software. Naïve WT mouse was used as negative control.

### Mass spectrometric protein identification

Label-free quantification by mass spectrometry was performed at the Mass Spectrometry Technology Access Center at the McDonnell Genome Institute (MTAC@MGI).

The aortic tissues (n = 4 non-treated, NT and n = 4 treated, T) from TGF-β blockade model of AAA were harvested on day 14 and homogenized. Concentration of protein lysate was measured by BCA assay and 400 µg of total protein per sample were purified by the acetone/TCA precipitation. The proteins were reduced, alkylated, and digested with trypsin according to the optimized protocol. Digested peptides were desalted on C18 spin columns. After colorimetric quantification, equal mass of digested peptides was used for label-free quantification by mass spectrometry. All samples were analyzed on MS in technical duplicates by randomization. For each technical replicate, the LFQ value derived from the MS intensity was calculated from the area under the curve. The results were formatted with the standard output for LFQ analysis. Protein was filtered for >1 unique peptide, which yielded a total of 3467 unique proteins.

### Proteomic data exploration, analysis and visualization

Analysis without imputation. For each mouse peptide, we calculated the estimated fold-change following treatment and statistical significance using a linear mixed model. We used nlme package (v3.1-163) in the R programming language (v4.3.1). The response was log2 of the LFQ values, status (treated or not) was a fixed-effect predictor, and biological sample name and technical replicates were modeled as random effects. We extracted the estimated log2 fold-change of status and the associated p-values using the broom package (broom v1.0.5; broom.mixed v0.2.9.4). No changes were significant using strict Bonferroni p-value correction or Benjamini-Hochberg FDR. There were 97 proteins with nominally significant p-values, which we used for further analysis. We used the g:ProfileR package (gprofiler2 v 0.2.2) to separately calculate enrichment of proteins with increased and decreased detection with treatment 63. In each case, we used the protein's associated gene names as input, *M. musculus* as the query organism, not an ordered query, not a multiquery, excluding electronic annotation, p-value correction with gSCS, minimum term size of 2, and maximum term size of 500. We also used the nominally significant genes (log2 transformed LFQ values) for principal components analysis (PCA) to assess the similarity among samples overall.

Analysis with imputation. The missing data for proteins expressed in at least two samples within a group were imputed based on normal distribution within that group, utilizing the R mouse package (doi:10.18637/jss.v045.i03). Proteins expressed in fewer than one sample within a group were excluded from the analysis. Gene Set Enrichment Analysis (GSEA) was carried out using the R FGSEA package (bioRxiv. doi:10.1101/060012). Single-sample pathway enrichment analysis was performed using the R package Singscore (citation doi:10.1093/nar/gkaa802). Visualization of results was accomplished using R packages pheatmap and ggplot2.

### Other statistical analysis

Comparisons between two groups of mice were performed by two-tailed, unpaired t-test without correction. Comparisons between multiple groups (≥ 3) were performed by one-way ANOVA followed by Bonferroni's post-test to compare all groups of data. F test was used to compare variances within each group of data and the difference in variances was found to be not significant between groups of animals. Data are presented as the mean ± SEM. A *p* value < 0.05 was considered significant.

## Supplementary Material

Supplementary figures and tables.

## Abbreviations

AAA: abdominal aortic aneurysm
AD: aortic diameter
HA: hyaluronic acid
IFN: interferon
IL: interleukin
MMP: matrix metalloprotease
NOS: nitric oxide synthase
ROS: reactive oxygen species
iNOS: inducible nitric oxide synthase
SOD: superoxide dismutase
NO: nitric oxide
NP: nanoparticle
TGF-β: transforming growth factor-β
siRNA: small interfering RNA
TNF: tumor necrosis factor
TUNEL: terminal deoxynucleotidyl transferase dUTP and nick labeling
TEM: transmission electron microscope
DLS: dynamic light scattering
PALS: Phase Analysis Light Scattering
FBS: fetal bovine serum
MEM: Minimum Essential Medium
VVG: Verhoeff-van Gieson
PCA: Principal Component Analysis
GSEA: Gene Set Enrichment Analysis
miR: microRNA
TCA: tricarboxylic acid
H_2_O_2_: hydrogen peroxide
PKC: protein kinase C
GAP: GTPase-activating protein
SMC: smooth muscle cell
