# Supplementary material for: Augmented expression of superoxide dismutase 2 mitigates progression and rupture of experimental abdominal aortic aneurysm
Source: Theranostics. 2025 Mar 10;15(9):4016–32. doi: 10.7150/thno.104957 (PMC11980667; doi:10.7150/thno.104957)
Supplement: Supplementary file 1 — Supplementary figures and tables. [file thnov15p4016s1.pdf]

Augmented expression of superoxide dismutase 2 mitigates progression and rupture of experimental abdominal aortic aneurysm

Huimin Yan[1,2]*, Ying Hu[1,2], Yang Lyu[3], Antonina Akk[1], Angela C. Hirbe[3], Samuel A. Wickline[1], Hua Pan[1], Elisha D.O. Roberson[1], and Christine T.N. Pham[1,2]*

Supplemental Figures

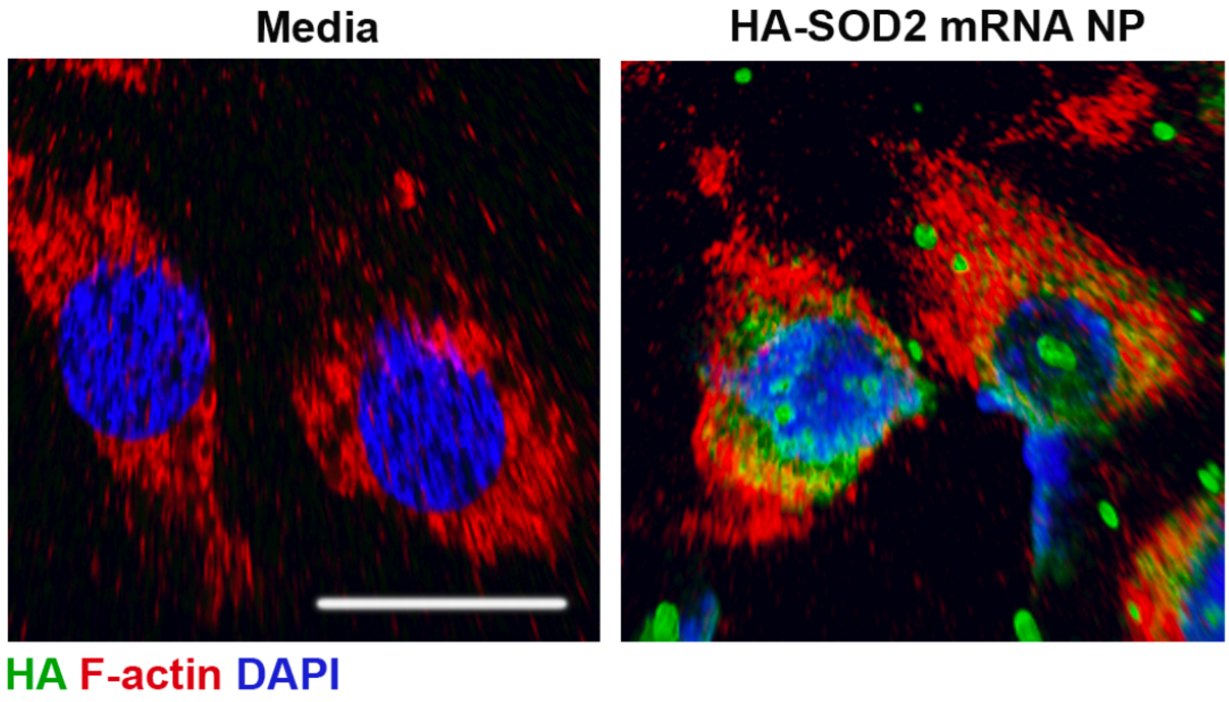

Figure S1. Uptake of HA-SOD2 mRNA NP *in vitro*. Bone-marrow-derived macrophages were left media or transfected with fluorescein-labeled HA-SOD2 mRNA NP for 4 hours then analyzed by confocal microscopy. NP = green; F-actin = red; DAPI (blue) stains nuclei. Scale bar = 20 μm

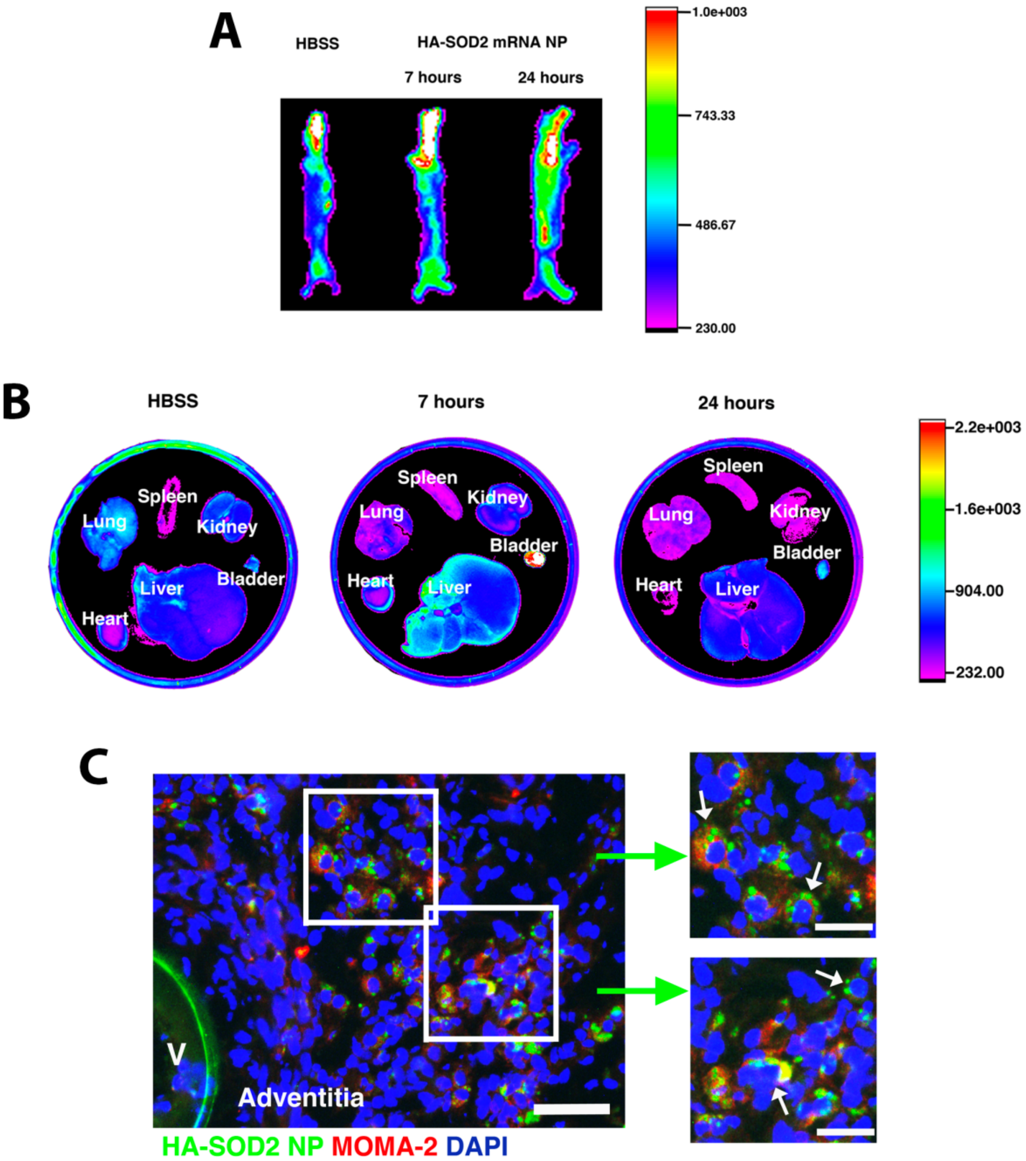

Figure S2. In vivo uptake of HA-SOD2 mRNA NP. (A) Elastase-perfused mice were injected i.v. with HBSS or fluorescein-HA-SOD2 mRNA NP on day 9 and IVIS of aortas were obtained 7 and 24 hours after injection. (B) Intensity of NP fluorescence in major organs at 7 and 24 hours after fluorescein-HA-SOD2 mRNA NP injection. (C) Mice were perfused with elastase on day 0, administered fluorescein-HA-SOD2 mRNA NP i.v. on day 9 post-elastase perfusion (mRNA= 1 μg per treatment) and fluorescence assessed in aortic tissue at 24 hours after injection. Aortic sections were examined for NP (green) and MOMA-2 (red). Colocalization appears yellow (arrow). DAPI (blue) stains nuclei. Scale bars = 50 μm, insert box = 25 μm.

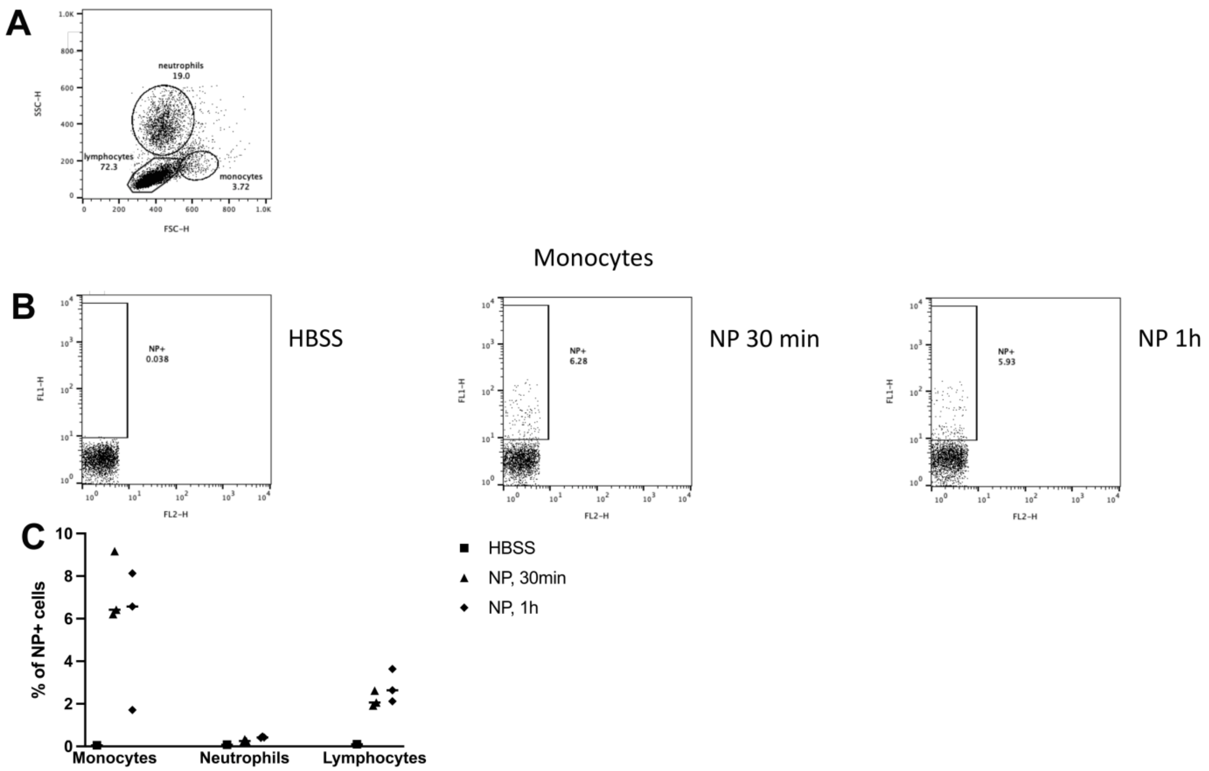

Figure S3. Uptake of HA-SOD2 mRNA NP in the circulation. Mice were injected i.v. with HBSS or fluorescein-labeled HA-SOD2 mRNA NP on day 9 post-elastase perfusion and sacrificed 30 min or 1 h after injection. Blood was obtained, RBC lysed, and WBC analyzed by flow cytometry. (A) Cell types were identified by size and granularity. (B) NP can be seen internalized by a small percentage of monocytes at both time points. (C) Quantification of NP internalization in different cell populations.

Table 1 Hematologic parameters in mice treated with nanoparticles

Treatment / Parameters	HBSS control	HA coated Scrambled NPs	HA coated SOD2 mRNA NPs
WBC (10^3/μl)	8.718 ± 0.386	9.395± 1.232	7.545 ± 1.569
RBC (10^6/μl)	8.743 ± 0.453	9.115 ± 0.193	8.375 ± 0.712
HGB (g/dL)	12.18 ± 0.230	11.10 ± 0.286	10.48 ± 0.966
Platelet (10^3/μl)	451.8 ± 143.7	456.6 ± 173.4	469.8 ± 164.4
HCT (%)	43.58 ± 2.070	41.03 ± 0.927	41.93 ± 0.904
MCV	47.25 ± 1.164	45.00 ± 0.385	44.80 ± 0.593
MCH	13.84 ± 0.316	12.15 ± 0.096	12.63 ± 0.384
MCHC	29.26 ± 1.396	27.05 ± 0.132	27.60 ± 0.584

Table 2 Chemical parameters in mice treated with nanoparticles

Treatment / Parameters	HBSS control	HA coated Scrambled NPs	HA coated SOD2 mRNA NPs
BUN (mg/dL)	32.06 ± 1.590	30.90 ± 2.465	32.93 ± 1.275
Creat (mg/dL)	0.347 ± 0.037	0.338 ± 0.009	0.339 ± 0.024
ALKP (U/L)	74.70 ± 6.533	73.02 ± 7.407	67.61 ± 3.585
AST (U/L)	48.31 ± 3.820	48.80 ± 7.017	49.17 ± 5.281
ALT (U/L)	38.73 ± 10.59	41.80 ± 9.272	42.99 ± 10.96

Figure S4. Toxicity profiles of NP following repeated (x3) injections

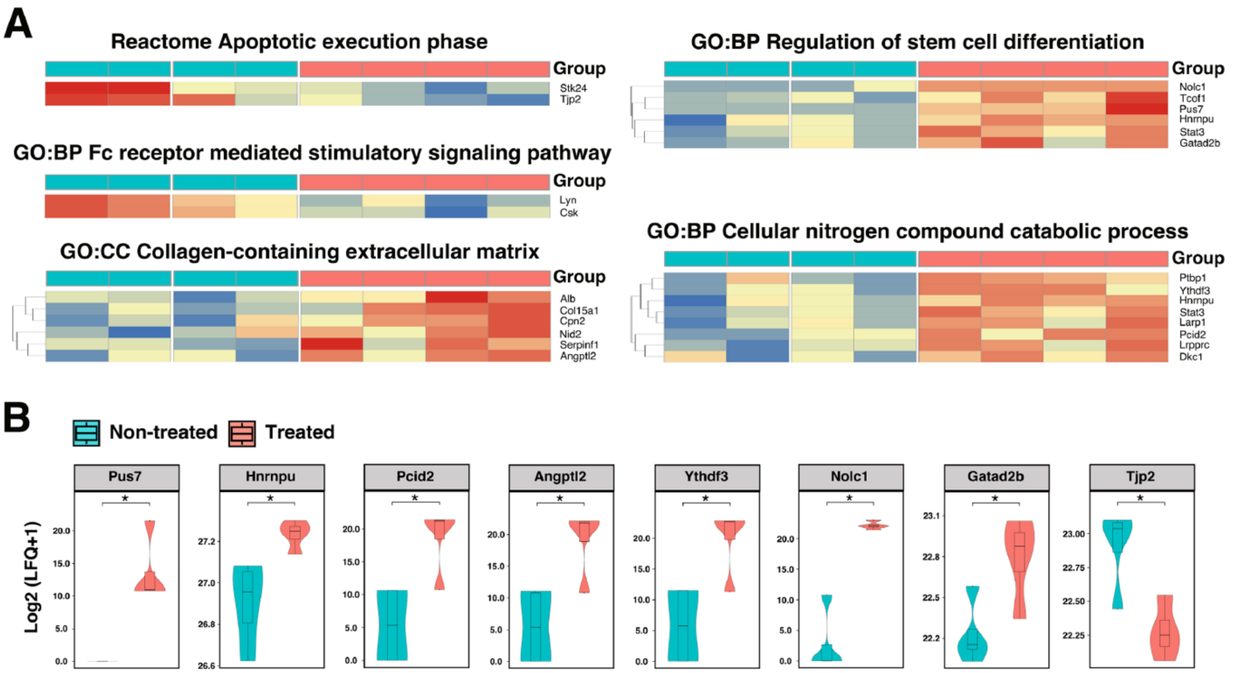

Figure S5. Proteomic profiling in TGF-β blockade model of AAA following HA-SOD2 mRNA NP administration. (A) Heatmaps of significantly enriched pathways. (B) Enhancement of key protein components following HA-SOD2 mRNA NP treatment. *P < 0.001.

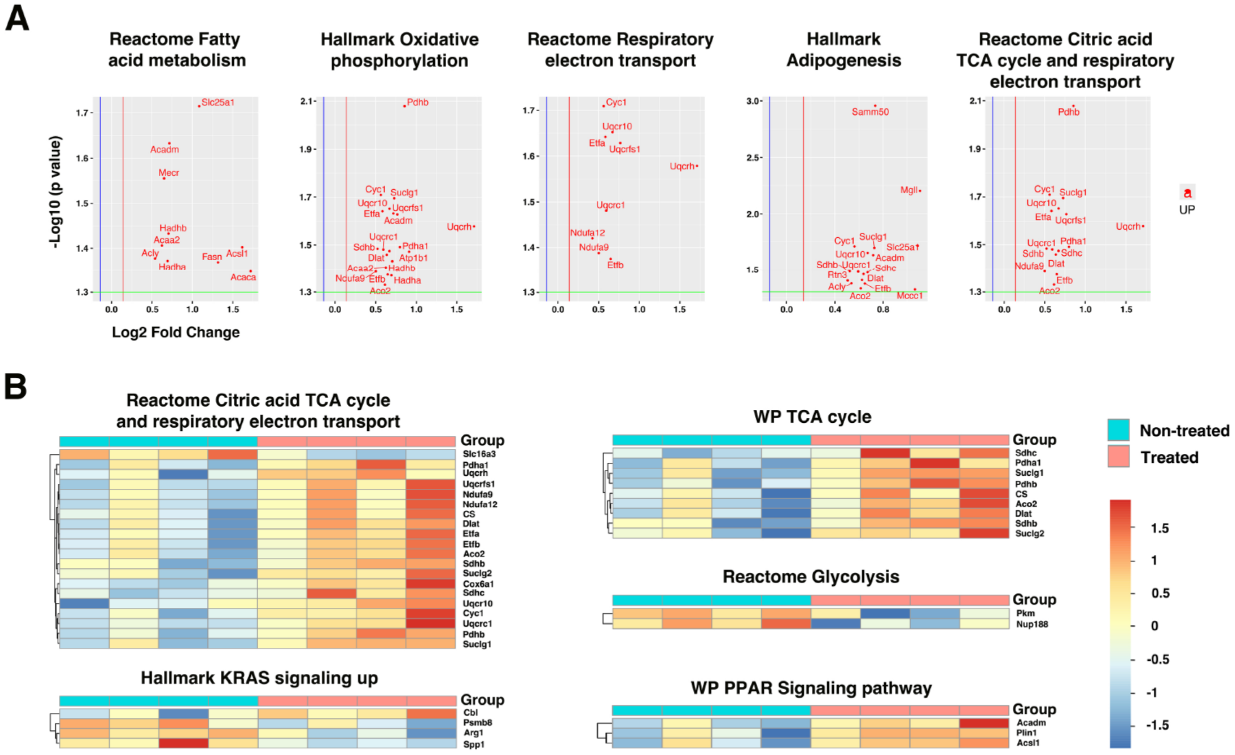

Figure S6. Contribution of SOD2 in the maintenance of mitochondrial redox balance. (A) Volcano plot of differential expressed proteins ($p<0.05$ and fold change >1.1) for each pathway after HA-SOD2 mRNA treatment. (B) GSEA and heatmaps of significantly enriched pathways in mitochondria following SOD2 augmentation in TGF-β blockade model of AAA.
